# Digital Evaluation of Aroma Intensity and Odor Characteristics of Tea with Different Types—Based on OAV-Splitting Method

**DOI:** 10.3390/foods11152204

**Published:** 2022-07-25

**Authors:** Wenwen Hu, Gege Wang, Shunxian Lin, Zhijun Liu, Peng Wang, Jiayu Li, Qi Zhang, Haibin He

**Affiliations:** 1 Key Laboratory of Agroecological Processing and Safety Monitoring of Fujian Province, Fujian Agriculture and Forestry University, Fuzhou 350002, China; 2180525002@fafu.edu.cn (W.H.); 1200525024@fafu.edu.cn (G.W.); 2200525005@fafu.edu.cn (S.L.); 1200525017@fafu.edu.cn (Z.L.); 2210101003@fafu.edu.cn (P.W.); ljy@fafu.edu.cn (J.L.); zhangqi1113@126.com (Q.Z.); 2 College of Tea and Food Science, Wuyi University, Wuyishan 353400, China

**Keywords:** tea, odor activity value (OAV), OAV-splitting method, odor characteristic, odor index I(o)

## Abstract

Aroma is one of the most important quality indicators of tea. However, this evaluation method is a subjective one. In this study, the volatiles of tea with 5 types were determined by headspace solid-phase micro-extraction (HS-SPME) combined with gas chromatography mass spectrometry (GC-MS). The aroma intensity and odor characteristics of teas were comparatively analyzed based on the OAV-splitting method. The results showed that OAV were green tea (492.02), red tea (471.88), oolong tea (302.74), white tea (68.10), and dark tea (55.98). The odor index I(o) indicated that green tea was strong-flavor tea with highlight green accompanied by fruity, woody and fatty odors; oolong tea was strong-flavor tea with fruity and fatty accompanied by woody, floral and green odors; red tea was strong-flavor tea with highlight fruity accompanied by woody, green and floral odors; white tea was a light-flavor tea with floral, woody and green odors; and dark tea was light-flavor tea with woody and floral notes accompanied by fatty and green odors. These results fitted perfectly with the people’s consensus on these teas, and proved that the OAV-splitting method is feasible to evaluate the aroma intensity and odor characteristics of tea aroma. We suggest that the digital evaluation of tea aroma can facilitate people’s communication.

## 1. Introduction

Chinese tea is conventionally classified into six types, i.e., white tea, green tea, red tea, oolong tea, dark tea, and yellow tea. Each type has distinct characteristics in appearance and color of leaves, and color, taste and aroma of its infusion [1]. Taste and aroma are the most important quality indicators of tea. In China and other countries, the quality and grade of tea are generally evaluated by tea appraisers according to an evaluation index system. Aroma usually accounts for 35% of the evaluation index of various teas, making it the most important factor of tea sensory quality [2]. The aroma quality directly affects the consumer interpretation of tea quality.

The aroma of tea mainly comes from the olfactory sense produced by volatile compounds, including the physical and mental stimulation brought on by the aroma compounds [3]. Aroma is the result of the combination of different aroma compounds at specific concentrations and proportions, in conjunction with their comprehensive effects at the olfactory nerves [4]. The aroma compounds account for only about 0.01% of the dry tea mass. Although the proportion is very low, the sensory threshold of aroma and its value impart key qualities favored by consumers [5]. About 600 aroma compounds have been identified in teas, including more than 280 compounds in green tea and more than 300 compounds in red tea and oolong tea [4,5,6,7,8]. According to the organic classification method, aroma compounds in tea can be classified into terpenoids, alcohols, aldehydes, ketones, acids, esters, heterocycles, and others [1,2,3]. How to evaluate the aroma quality of tea through so many aroma compounds has always been a problem. As early as the 1990s, Owuor proposed the terpenoid index (TI) to classify and evaluate teas and used the TI to evaluate the aroma quality of teas [9]. The TI has not been widely used and popularized, as only the content of terpenoids is used to evaluate the quality of tea. Recent studies have shown that the intensity and characteristics of aroma cannot be evaluated solely based on the quantity and content of aroma compounds. In fact, compounds of high contents do not necessarily produce a strong aroma. All types of tea usually contain dozens or even hundreds of volatile compounds, yet not all volatile components play a dominant role in the aroma quality of tea. Moreover, they do not have a linear relationship with compound concentration. Wang et al. [10] showed that only a few compounds determined the overall aroma quality of tea. When some key compounds are missing, the overall aroma characteristics of tea are significantly affected, and this has been verified in scented teas [6]. The compounds that are essential for the formation of aroma characteristics of tea are termed key aroma components [11]. The concept of odor activity value (OAV) was proposed by Patton and Josephson [12], and it is defined as the ratio of the content of an aroma compound to the threshold of the compound. Normally, when OAV > 1, the compound has a significant contribution to the overall aroma characteristics [11]. The overall aroma characteristics of food are related to the content of the individual compounds that make up the food, as well as the threshold of the compound [10,11,12]. The OAV method is an important strategy for screening key aroma components in foods, and it has been used for the analysis of cheese, french fries, rice wine, and tobacco [13,14,15,16]. In addition, the aroma composition of a compound is complex, and a single odor cannot characterize the entire aroma of a compound. For example, benzyl acetate has 70% jasmine odor, 20% fruity odor, and 10% chemical odor [17]. Standardized quantitative terms, rather than vague words such as “intense floral and fruity aroma “ and “this tea is more fruity and woody than that one”, should be used to describe the aroma characteristics so that the aroma profiles and intensities of different products can be objectively and quantitatively compared [1]. This practice has been widely used in adjusting the aroma of various products, such as beverages, champagne, cigarettes, cosmetics, and daily flavor and fragrance industry. 

Currently, studies on the aroma characteristics of various types of tea mainly focus on the comparative analysis of their components and contents [18,19,20,21]. A systematic investigation on the aroma intensity and odor characteristics of tea, especially the objective digital evaluation using the OAV method, is lacking. In this study, five types of teas were analyzed using the OAV-splitting method to identify the aroma intensity and odor characteristics of different types of teas. This study will provide a digital evaluation method for tea aroma which can better and more accurately communicate with consumers, dealers and appraisers. 

## 2. Materials and Methods

### 2.1. Tea Samples and Chemicals

Tea samples were purchased from the tea market and kept in a sealed refrigerator at 4 °C. White tea (Bai Hao Yin Zhen) was a product of Fujian Fuding White Tea Co., LTD which was located in Fuding City, Fujian Province, China. Green tea (Xihu Longjing) was a product of Zhejiang Tianfu Tea Co., Ltd which was located in Shaoxing City, Zhejiang Province, China. Oolong tea (Tie Guan Yin) came from the Fujian Huaxiangyuan Tea Industry Co., Ltd which was located in Fuzhou City, Fujian Province, China. The red tea (Lapsang Souchong) was made by the Fujian Wuyi mountain Tongmuguan GuoHong Tall Tea Co., Ltd which was located in Wuyishan City, Fujian Province, China. Dark tea (Anhua Dark Tea) was procured from the Hunan Jiuyang Tea Co., Ltd which was located in Anhua City, Hunan Province, China.

The mixed standard solution of n-alkanes (C_10_–C_20_ even number) was o2si smart solutions A, which was purchased from the LGC Standards Company (Charleston, SC, USA). A solid-phase micro extraction (SPME) needle (50/30 μm, DVB/CAR/PDMS) was purchased from Supelco (Bellefonte, PA, USA). 

### 2.2. Extraction and Analysis of Tea Volatile Compounds

The extraction and analysis of tea volatile compounds were carried out by headspace solid-phase micro-extraction (HS-SPME) combined with gas chromatography mass spectrometry (GC-MS). The tea samples were removed from the 4 °C refrigerator and brought to room temperature for 1 h. The samples were ground using a mortar and pestle, and the powder was collected after passing through a 60 mesh (0.250 mm) filter. Two grams of the powder sample was added to a 20 mL headspace bottle. The bottle was then sealed and placed in a 50 °C heater for 30 min to equilibrate and fully volatilize the tea volatile compounds. The SPME needle (aged for 1 h before the test) was inserted into the headspace bottle to absorb for 30 min. The needle was then immediately inserted into the GC-MS injection portal and desorbed for 5 min. Each sample was analyzed three times in parallel. 

The GC-MS instrument was a 7890A-5975C GC-MS (Agilent, USA). GC conditions were as follows: column model (DB-WAXetr 60 m × 0.32 mm × 0.25 μm); Helium (purity >99.999%) was carrier gas with flow rate of 1 mL/min. The temperature procedure was as follows: it was raised from 35 °C (held for 5 min) to 150 °C at a rate of 3 °C/min, then raised to 240 °C at 10 °C/min and held for 2 min. The MS conditions were as follows: EI mode, ionization energy was 70 eV; emission current was 34.6 μA. The ion source temperature was 230 °C; the quadrupole temperature was 150 °C; and the interface temperature was 240 °C. The mass scan range was *m*/*z* 45–500 amu. 

### 2.3. Qualitative and Quantitative Analysis of Tea Aroma Components

The volatile compounds were identified by comparing the mass spectra in the National Institutes of Standards and Technology Mass Spectrometry Library (NIST8.0), combined with the retention indices, the similarity match and the relative abundance of fragment ions. The n-alkanes standard (C_10_–C_20_) as the external standard was injected under the same GC-MS conditions as the sample. The contents of the volatile compounds were calculated and reported on the basis of the external standard concentration. 

### 2.4. Calculation of OAV of Teas and Analysis of Odor Characteristics

The threshold values of compounds were referenced in the literature (as shown in Supplementary Table S1) [22,23]. The OAV of each volatile compound (OAVi) was calculated as OAVi = Ci/Ti, where Ci is the content of compound i (μg kg^−1^), and Ti is the threshold value of compound i (μg kg^−1^). The compounds with OAVi ≥ 1 were identified as the odor-active compounds for further comparative analysis. The total OAV of a tea (OVAt) is the sum of those OAVi of the odor-active compounds, namely OVAt = ∑OAVi. The OAVt represent the aroma intensity of a tea. 

Based on the reference (Table S1) [17], the OAVi of a compound were split into six odor characteristics, i.e., woody, floral, burnt, green, fruity, and fatty. Next, the OAV values of each odor characteristic in a tea were obtained. The index of each odor characteristic, namely I(o), is the ratio of the OAV value of each odor characteristic to the OAVt of tea. I(o) represents the intensity of an odor. The intensity and characteristic of each odor is OAVt × I(o). 

## 3. Results and Discussion

### 3.1. Analysis of Volatile Compounds in Tea with Different Types

Volatile compounds in five tea types were shown in Supplementary Table S2. The results showed that 39 compounds (32.01 μg kg^−1^) were found in white tea, including six terpenoids, seven alcohols, four aldehydes, three ketones, eight acids, four esters, four heterocycles, and three hydrocarbons. Fifty-eight compounds (33.88 μg kg^−1^) were found in green tea, including 12 terpenoids, 10 alcohols, six aldehydes, eight ketones, five acids, five esters, eight heterocycles, and four hydrocarbons. Fifty-two compounds (33.23 μg kg^−1^) were found in oolong tea, including 14 terpenoids, six alcohols, nine aldehydes, five ketones, three acids, two esters, six heterocycles, and seven hydrocarbons. Fifty-four compounds (76.60 μg kg^−1^) were found in red tea, including 11 terpenoids, seven alcohols, seven aldehydes, four ketones, six acids, five esters, eight heterocycles, and six hydrocarbons. Forty compounds (25.48 μg kg^−1^) were found in dark tea, including nine terpenoids, nine alcohols, seven aldehydes, three ketones, three acids, two esters, two heterocycles, and five hydrocarbons. 

In terms of the number, it was green tea (58) > red tea (54) ≈ oolong tea (52) > dark tea (40) ≈ white tea (39). In terms of content (μg kg^−1^), it was red tea (76.60) > green tea (33.88) ≈ oolong tea (33.23) ≈ white tea (32.01) > dark tea (25.48). Many compounds, such as terpenoids (linalool and oxides, β-ionone, β-cyclocitral, dihydroactindiolide), alcohols (1-penten-3-ol, (*E*)-3-hexen-1-ol, benzyl alcohol, phenethyl alcohol), aldehydes and ketones (hexanal, (*E*)-2-hexenal, (*E*,*E*)-2,4-heptadienal, benzaldehyde, 6-methyl-5-hepten-2-one), acids and esters (acetic acid, propionic acid, hexanoic acid, methyl salicylate), and heterocycle compounds (2-pentylfuran and 2-acetyl pyrrole), were widely reported as tea aroma compounds [20]. It is apparent and impossible to clearly define the aroma intensity and odor characteristic of tea with different types depended on the number and the contents of volatile compounds in Table S2. Only a compound with OAV > 1 has its contribution to the aroma of a product [12]. The higher the OAV of a compound, the more the contribution to the overall aroma of a product. The overall aroma intensity of a product are related to the content and the threshold of the individual compounds [10]. The OAV method is widely used for defining the aroma intensity and odor characteristic in foods, wine and tobacco [13,14,15,16]. Therefore, we calculated the OAVi of each compound to analyze the key contributors for tea aroma in the subsequent section. 

### 3.2. Analysis of OAV, Odor Characteristic and Key Compounds in Tea with Different Types

There were eight compounds with OAVi > 1 in white tea and the OAVt was 68.10 (Table 1). They were β-ionone (22.86) >> (*E*)-3-hexen-1-ol (10.43) > 6-methyl-5-hepten-2-one (9.40) > methyl salicylate (6.75) ≈ linalool (6.58) > Epoxydihydrolinalool (5.82) > phenethyl alcohol (4.51) > hexanal (1.75). The OAVi of the first three compounds was much higher than that of other compounds, accounting for 62.69% of the OAVt. 

In terms of odor characteristics, the OAVt (68.10) of white tea was composed of 28.36 for floral, 17.71 for woody, 10.01 for green, 6.59 for burnt, 4.21 for fruity, and 1.22 for fatty. The floral odor (accounting for 41.64%) was the first feature of white tea, complemented by woody (accounting for 26.01%), as well as green (accounting for 14.70%). These three odors (accounting for 82.35% of OAVt) formed the weak fragrance of white tea with floral odor as dominance, hierarchically mixing odors of floral, wood, and green. 

In terms of aroma compounds in white tea, β-ionone, 6-methyl-5-hepten-2-one and linalool were the main and even contributors for floral odor; β-ionone was the first contributor for woody odor, secondary by epoxydihydrolinalool; and (*E*)-3-hexen-1-ol was the first and dominant contributor for green odor. 

There were 13 compounds with OAVi > 1 in green tea and the OAVt was 492.02 (Table 2). They were 3-octanone (230.00) >> β-cyclocitral (100.00) >> valeraldehyde (55.83) >> β-ionone (27.14) > (*E*)-3-hexen-1-ol (22.00) > 6-methyl-5-hepten-2-one (14.20) ≈ 1-penten-3-ol (14.07) > 1-octen-3-ol (13.00), as well as five compounds (<5.3). The OAVi of the first three compounds was much higher than that of other compounds, accounting for 78.41% of the OAVt. 

In terms of odor characteristics, the OAVt (492.02) of green tea was composed of 224.09 for green, 116.84 for fruity, 58.36 for woody, 52.95 for fatty, 21.46 for burnt, and 18.32 for floral. The green odor (accounting for 45.55%) was the first feature of green tea, complemented by fruity (accounting for 23.75%), as well as woody (accounting for 11.86%) and fatty (accounting for 10.76%). These four odors (accounting for 91.92% of OAVt) formed the strong fragrance of green tea with green odor stand out, fruity odor obvious, accompanied by odors of softly woody and fatty. 

In terms of aroma compounds in green tea, 3-octanone was the first and dominant contributor for green odor; β-cyclocitral was the first contributor for fruity odor, secondary by 3-octanone; 3-octanone was the first contributor for woody odor, secondary by β-ionone and β-cyclocitral; and valeraldehyde was the first and dominant contributor for fatty odor. 

There were 10 compounds with OAVi > 1 in oolong tea and the OAVt was 302.74 (Table 3). They were β-cyclocitral (120.00) >> valeraldehyde (66.67) > β-ionone (45.71) > 6-methyl-5-hepten-2-one (30.80) > (*E*,*E*)-2,4-heptadienal (14.49), as well as five compounds (<7.4). The OAVi of the first four compounds was much higher than that of other compounds, accounting for 86.93% of the OAVt. 

In terms of odor characteristics, the OAVt (302.74) of oolong tea was composed of 111.04 for fruity, 66.74 for fatty, 35.55 for woody, 35.27 for floral, 34.73 for green, and 19.40 for burnt. The fruity odor (accounting for 36.68%) was the first feature of oolong tea, complemented by fatty (accounting for 22.05%), as well as woody (accounting for 11.74%), floral (accounting for 11.65%), and green (accounting for 11.48%). These five odors (accounting for 93.60% of OAVt) formed the strong and the most abundant fragrance of oolong tea with fruity odor as the dominant one and fatty odor as complementary, consisting of aromas of flowers, wood, and green. 

In terms of aroma compounds in oolong tea, β-cyclocitral was the first and dominant contributor for fruity odor; valeraldehyde was the first contributor for fatty odor, secondary by (*E*,*E*)-2,4-heptadienal; β-ionone and β-cyclocitral were the main contributors for woody odor; 6-methyl-5-hepten-2-one and β-ionone were the main contributors for floral odor; and β-cyclocitral was the first and dominant contributor for green odor.

There were 13 compounds with OAVi > 1 in red tea, and the OAVt was 471.88 (Table 4). They were β-cyclocitral (263.33) >> β-ionone (94.29) >> 6-methyl-5-hepten-2-one (36.60) > (*E*)-3-hexen-1-ol (21.71) > furfural (16.38) > 1-pentanol (10.71) ≈ methyl salicylate (10.25), as well as six compounds (<4.4). The OAVi of the first two compounds was much higher than that of other compounds, accounting for 75.78% of the OAVt. 

In terms of odor characteristics, the OAVt (471.88) of red tea was 202.19 for fruity, 74.14 for woody, 72.93 for green, 66.49 for floral, 35.47 for burnt, and 20.66 for fatty. The fruity odor (accounting for 42.85%) was the first feature of red tea, complemented by woody (accounting for 15.71%), green (accounting for 15.45%), and floral (accounting for 14.09%). These four odors (accounting for 88.10% of OAVt) formed the strong and abundant fragrance of red tea with distinctive fruity odor, compositing evenly odors of wood, green, and floral. 

In terms of aroma compounds in red tea, β-cyclocitral was the first and dominant contributor for fruity odor; β-ionone was the first contributor for woody odor, secondary by β-cyclocitral; β-cyclocitral was the first contributor for green odor, secondary by (*E*)-3-hexen-1-ol; and β-ionone and 6-methyl-5-hepten-2-one were the main contributors for floral odor.

There were 10 compounds with OAVi > 1 in dark tea and the OAVt was 55.98 (Table 5). They were β-ionone (34.29) >> 6-methyl-5-hepten-2-one (6.40) > (*E*,*E*)-2,4-heptadienal (5.71), as well as 7 compounds (<1.7). The OAVi of the first three compounds was much higher than that of other compounds, accounting for 82.88% of the OAVt. 

In terms of odor characteristics, the OAVt (55.98) of dark tea was composed of 18.37 for woody, 17.15 for floral, 9.55 for fatty, 7.50 for burnt, 2.96 for fruity, and 0.45 for green. The odors of woody (accounting for 32.81%) and floral (accounting for 30.64%) were evenly matched in the characteristics of black tea, complemented by fatty (accounting for 17.06%). These three odors (accounting for 80.51% of OAVt) formed the weak fragrance of dark tea with odors of woody and floral, compositing of mild fatty. 

In terms of aroma compounds in dark tea, β-ionone was the first and dominant contributor for woody, floral and burnt odors; (*E*,*E*)-2,4-heptadienal was the first and dominant contributor for fatty odor. 

It is well-known that the number and the content of tea volatile compounds could not accurately represent the aroma quality of tea. The result of OAVt of the five tea samples was green tea (492.02) ≈ red tea (471.88) > oolong tea (302.74) >> white tea (68.10) ≈ dark tea (55.98) (Table 1, Table 2, Table 3, Table 4 and Table 5). These results suggested that green, red, and oolong teas are high-aroma teas, while white tea and black tea are low-aroma types. This conclusion is surprisingly consistent with the sensory cognition of the general public on these five types of tea, that is, green, red, and oolong teas are considered strong-flavor tea and white and black teas are mild-flavor teas [24]. More and more studies have reported on the tea aroma characteristics by using the OAV method. Gong et al. used the OAV method to study the aroma characteristics of Longjing tea (a green tea) and found that the OAV of geraniol, epoxylinalool, (*Z*)-nerolidol, 2-ethyl-3,5-dimethylpyrazine, and β-ionone were relatively high [25]. Wang et al. reported that 14 compounds were the key aroma compounds of Longjing tea based on the gas chromatography-olfactometry (GC-O), OAV method and an aroma recombination experiment [10]. Zhu et al. reported that 24 compounds (OAV > 1) were identified as important compounds and were contributors to the overall aroma of Laoshan green teas [26]. Nie reported that the compounds with OAV > 1 were 29 in green tea, 31 in red tea, and 29 in flower tea. Ten compounds in green tea showed green odor, five compounds in red tea showed baking odor, and 23 compounds and 19 compounds in flower tea showed fruit and floral odors [27]. However, the OAVt of tea can only compare the difference of aroma intensity between tea samples, but can not distinguish the different aroma types, as well as the intensity of various odors. For example, green odor is the most characteristic odor of green tea, no matter what green tea samples. Almost all red tea worldwide has a strong fruity odor. Therefore, the OAVt of tea tells us nothing about the characteristic odor of tea. Currently, when people are discussing about tea fragrance such as ‘the tea aroma of A is much intense than that of B’, and ‘the fruity odor of No 1 tea sample is better than that of No 2′, how much is that ‘much intense’ and ‘better than’? Tea aroma is a complex of various odors such as woody, floral, burnt, green, fruity, and fatty. We need to split the OAVt of tea into various odors and then find the combination of those odors and the dominant odors. 

### 3.3. Analysis of Odor Index in Teas of Different Types

**Figure 1:**
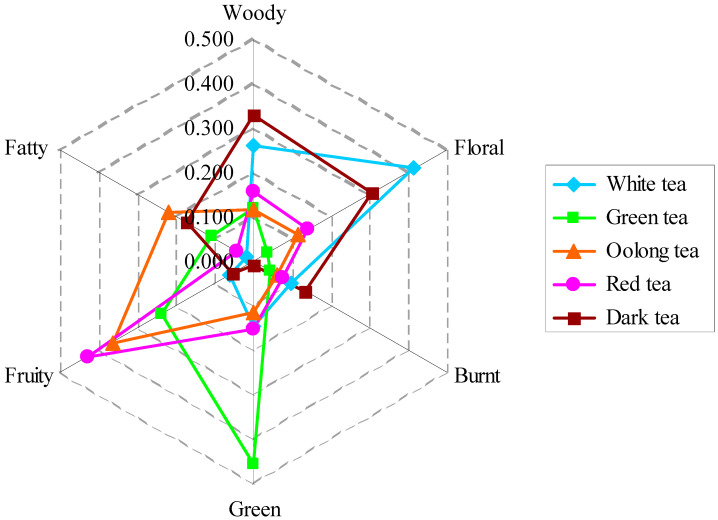
Radar chart of index of odor I(o) in tea with different types.

There is a general belief that a compound has a variety of odors and many compounds have a common odor. For example, β-ionone has a combination of woody, floral and burnt odors [28,29]. (*E*)-3-Hexen-1-ol has a strong green odor with light fruity odor [26,30,31], while β-cyclocitral has a strong fruity odor with light green and woody odors [17,32]. After the OVA-splitting process, the odor characteristic of each tea sample was clarified (Table 1, Table 2, Table 3, Table 4 and Table 5). For simplicity and application, the index of odor I(o) was introduced to describe the relative intensity of each odor to OAVt. The results showed that the odor characteristics of tea with different types have distinct differences (Figure 1). The aroma of white tea was mainly composed of I(floral) 0.416, I(woody) 0.260, and I(green) 0.147, and the sum of the three I(o) was 0.823. The aroma of green tea was mainly composed of I(green) 0.455, I(fruity) 0.237, I(woody) 0.119, and I(fatty) 0.108, and the sum of the four I(o) was 0.919. The aroma of oolong tea was mainly composed of I(fruity) 0.367, I(fatty) 0.220, I(woody) 0.117, I(floral) 0.117, and I(green) 0.115, and the sum of the five I(o) was 0.936. The aroma of red tea was mainly composed of I(fruity) 0.429, I(woody) 0.157, I(green) 0.154, and I(floral) 0.141, and the sum of the four I(o) was 0.881. The aroma of dark tea was mainly composed of I(woody) 0.328, I(floral) 0.306, I(fatty) 0.171, and I(green) 0.134, and the sum of the four I(o) was 0.939. 

**Figure 2:**
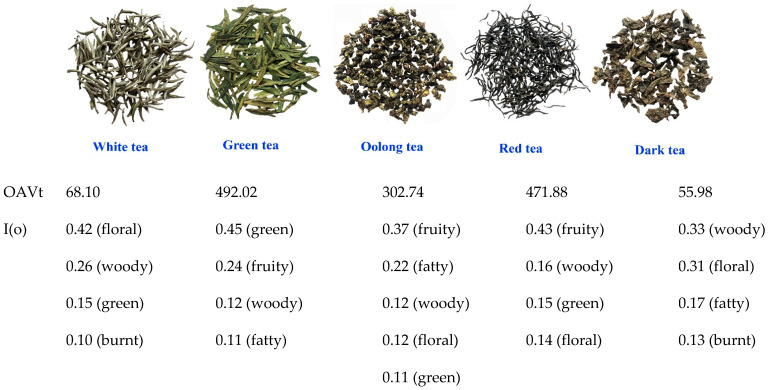
The OAVt and I(o) of tea with different types.

No any odor has its I(o) greater than 0.50 in five types of tea (Figure 1). This indicated that tea fragrance is complexed with one or two odors as dominant components, complemented by other odors. The difference of the dominant odor components in each type of tea determines the specific odor of the tea. In other words, the differential of the dominant odor components in teas contribute the differential of odor characteristic in each type tea. To briefly summarize, green tea is a strong-flavor tea (highest OAVt) with highlight green accompanied by fruity, woody, and fatty odors; oolong tea was strong-flavor tea (higher OAVt) with fruity and fatty accompanied by woody, floral, and green odors; red tea was strong-flavor tea (higher OAVt) with highlight fruity accompanied by woody, green, and floral odors; white tea was light-flavor tea (low OAVt) with floral, woody and green odors; and dark tea was light-flavor tea (low OAVt) with woody and floral accompanied by fatty and green odors. The results fitted perfectly with the people’s consensus on these five types of tea. Our results proved that the OAV-splitting method is feasible to evaluate the aroma intensity and odor characteristics of tea aroma. We hope that, in the near future, when people are discussing tea fragrance such as ‘the tea aroma of A is much more intense than that of B’, they could add ‘because the OAV of A is 492.02 and B is 68.10’ (Figure 2). Furthermore, subjective descriptions such as ‘the fruity odor of No 1 tea sample is better than that of No 2′ could also be updated to ‘No 1 tea sample is OAV = 471.88 and I(fruity) = 0.43, however, No 2 is OAV = 302.74 and I(fruity) = 0.37’ (Figure 2). The OAVt and I(o) are the digital evaluation for aroma quality of a tea. Therefore, the description and communication of tea fragrance will be more 

The aroma compounds, aroma intensity and odor characteristics of tea are also affected by many factors, such as variety and culturing, origin and region, processing and storage, and so on. It is difficult to consider the influence of various factors on tea aroma in this paper. In China, almost all of the central and southern province, as well as the western provinces, have green teas. Due to the feature of red tea technology, all tea varieties can be processed to red tea, even in the Henan and Guizhou provinces of China. Although only one tea sample of each type was selected for comparative analysis in this study, this does not affect the significance of our results for the digital evaluation of aroma intensity and the odor characteristic of tea. We suggested that digital evaluation of tea aroma will improve people to understanding aroma quality of tea, then help to bring about an open, fair and just market environment. 

## 4. Conclusions

In this paper, the OAV method combined with odor splitting was used to analyze the aroma intensity and odor characteristics of teas of different types. The results of OAVt showed that green, red, and oolong teas are strong-flavor types, while white and black teas are mild-flavor type, which are in full accord with people’ consensus on the aroma of these five teas. Further, the results of odor characteristics were surprisingly consistent with the sensory cognition of the general public on these five teas. These results supported that using objective digital language, such as OAVt and I(o), to define tea fragrance is a practicable solution that can better and more accurately communicate with consumers, dealers and appraisers. Our results provide a novel approach to evaluate the aroma quality of tea, which could make up for tea sensory evaluation. 

## Figures and Tables

**Table 1:** The content, OAVi and OAV-splitting of key aroma compounds in white tea.

Compound	Ci (μg kg^−1^)	OAVi	%	OAV-Splitting of Odor Characteristic					
				Woody	Floral	Burnt	Green	Fruity	Fatty
β-Ionone	0.16 ± 0.02	22.86	33.57	11.43	6.86	4.57	0	0	0
(*E*)-3-Hexen-1-ol	0.73 ± 0.01	10.43	15.32	0	0	0	9.39	1.04	0
6-Methyl-5-hepten-2-one	0.47 ± 0.01	9.40	13.80	0	6.58	0	0	2.82	0
Methyl salicylate	0.27 ± 0.02	6.75	9.91	0	4.73	2.02	0	0	0
Linalool	3.29 ± 0.08	6.58	9.66	0	6.58	0	0	0	0
Epoxydihydrolinalool	2.91 ± 0.26	5.82	8.55	5.65	0	0	0	0	0.17
Phenethyl alcohol	3.38 ± 0.56	4.51	6.62	0.45	3.61	0	0.45	0	0
Hexanal	0.84 ± 0.01	1.75	2.57	0.18	0	0	0.17	0.35	1.05
Total	12.05	68.10 *		17.71	28.36	6.59	10.01	4.21	1.22
% **				26.01	41.64	9.68	14.70	6.18	1.79

*Note*: OAVi = Ci/Ti, where Ci is the content of compound i (μg kg^−1^), and Ti is the threshold value of compound i (μg kg^−1^). * OVAt = ∑OAVi. ** % is proportion of the each odor OAV accounting for OAVt.

**Table 2:** The content, OAVi and OAV-splitting of key aroma compounds in green tea.

Compound	Ci (μg kg^−1^)	OAVi	%	OAV-Splitting of Odor Characteristic					
				Woody	Floral	Burnt	Green	Fruity	Fatty
3-Octanone	0.23 ± 0.01	230.00	46.74	34.50	0	0	172.50	23.00	0
β-Cyclocitral	0.30 ± 0.05	100.00	20.32	10.00	0	0	20.00	70.00	0
Valeraldehyde	0.67 ± 0.08	55.83	11.35	0	0	0	5.58	11.17	39.08
β-Ionone	0.19 ± 0.00	27.14	5.52	13.57	8.14	5.43	0	0	0
(*E*)-3-Hexen-1-ol	1.54 ± 0.07	22.00	4.47	0	0	0	19.80	2.20	0
6-Methyl-5-hepten-2-one	0.71 ± 0.06	14.20	2.89	0	9.94	0	0	4.26	0
1-Penten-3-ol	5.04 ± 0.25	14.07	2.86	0	0	5.63	2.81	5.63	0
1-Octen-3-ol	0.52 ± 0.04	13.00	2.64	0	0	10.40	1.30	0	1.30
1-Pentanol	0.79 ± 0.01	5.26	1.07	0	0	0	0	0	5.26
(*E*,*E*)-2,4-Heptadienal	0.25 ± 0.00	5.10	1.04	0	0	0	0	0	5.10
Hexanal	1.39 ± 0.02	2.90	0.59	0.29	0	0	0.29	0.58	1.74
2-Hexen-1-ol	0.31 ± 0.01	1.34	0.27	0	0	0	1.34	0	0
1-Octanol	0.13 ± 0.02	1.18	0.24	0	0.24	0	0.47	0	0.47
Total	12.07	492.02 *		58.36	18.32	21.46	224.09	116.84	52.95
% **				11.86	3.72	4.36	45.55	23.75	10.76

*Note*: OAVi = Ci/Ti, where Ci is the content of compound i (μg kg^−1^), and Ti is the threshold value of compound i (μg kg^−1^). * OVAt = ∑OAVi. ** % is proportion of the each odor OAV accounting for OAVt.

**Table 3:** The content, OAVi and OAV-splitting of key aroma compounds in oolong tea.

Compound	Ci (μg kg^−1^)	OAVi	%	OAV-Splitting of Odor Characteristic					
				Woody	Floral	Burnt	Green	Fruity	Fatty
β-Cyclocitral	0.36 ± 0.01	120.00	39.64	12.00	0	0	24.00	84.00	0
Valeraldehyde	0.80 ± 0.12	66.67	22.02	0	0	0	6.67	13.33	46.67
β-Ionone	0.32 ± 0.02	45.71	15.10	22.86	13.71	9.14	0	0	0
6-Methyl-5-hepten-2-one	1.54 ± 0.19	30.80	10.17	0	21.56	0	0	9.24	0
(*E*,*E*)-2,4-Heptadienal	0.71 ± 0.09	14.49	4.79	0	0	0	0	0	14.49
Furfural	2.06 ± 0.06	7.30	2.41	0	0	5.84	0	0	1.46
1-Penten-3-ol	2.59 ± 0.12	7.23	2.39	0	0	2.89	1.45	2.89	0
Hexanal	3.29 ± 0.10	6.87	2.27	0.69	0.00	0.00	0.69	1.37	4.12
(*E*)-3-Hexen-1-ol	0.15 ± 0.00	2.14	0.71	0	0	0	1.93	0.21	0
3,5-Octadien-2-one	0.23 ± 0.01	1.53	0.50	0	0	1.53	0	0	0
Total	12.05	302.74 *		35.55	35.27	19.40	34.74	111.04	66.74
% **				11.74	11.65	6.40	11.48	36.68	22.05

*Note*: OAVi = Ci/Ti, where Ci is the content of compound i (μg kg^−1^), and Ti is the threshold value of compound i (μg kg^−1^). * OVAt = ∑OAVi. ** % is proportion of the each odor OAV accounting for OAVt.

**Table 4:** The content, OAVi and OAV-splitting of key aroma compounds in red tea.

Compound	Ci (μg kg^−1^)	OAVi	%	OAV-Splitting of Odor Characteristic					
				Woody	Floral	Burnt	Green	Fruity	Fatty
β-Cyclocitral	0.79 ± 0.09	263.33	55.80	26.33	0	0	52.67	184.33	0
β-Ionone	0.66 ± 0.04	94.29	19.98	47.14	28.29	18.86	0	0	0
6-Methyl-5-hepten-2-one	1.83 ± 0.23	36.60	7.76	0	25.62	0	0	10.98	0
(*E*)-3-Hexen-1-ol	1.52 ± 0.02	21.71	4.60	0	0	0	19.54	2.17	0
Furfural	4.62 ± 0.41	16.38	3.47	0	0	13.10	0	0	3.28
1-Pentanol	1.61 ± 0.13	10.71	2.27	0	0	0	0	0	10.71
Methyl salicylate	0.41 ± 0.05	10.25	2.17	0	7.17	3.08	0	0	0
2(5H)-Furanone	0.26 ± 0.02	4.33	0.92	0	0.43	0.43	0	2.60	0.87
Acetic acid	22.57 ± 0.73	4.10	0.87	0	0	0	0	0.41	3.69
Hexanal	1.57 ± 0.01	3.28	0.70	0.33	0	0	0.32	0.66	1.97
Linalool	1.40 ± 0.10	2.80	0.59	0	2.80	0	0	0	0
Phenethyl alcohol	2.04 ± 0.22	2.72	0.58	0.27	2.18	0	0.27	0	0
Benzaldehyde	4.83 ± 0.71	1.38	0.29	0.07	0	0	0.13	1.04	0.14
Total	44.11	471.88 *		74.14	66.49	35.47	72.93	202.19	20.66
% **				15.71	14.09	7.52	15.45	42.85	4.38

*Note*: OAVi = Ci/Ti, where Ci is the content of compound i (μg kg^−1^), and Ti is the threshold value of compound i (μg kg^−1^). * OVAt = ∑OAVi. ** % is proportion of the each odor OAV accounting for OAVt.

**Table 5:** The content, OAVi and OAV-splitting of key aroma compounds in dark tea.

Compound	Ci (μg kg^−1^)	OAV	%	OAV-Splitting of Odor Characteristic					
				Woody	Floral	Burnt	Green	Fruity	Fatty
β-Ionone	0.24 ± 0.01	34.29	61.25	17.14	10.29	6.86	0	0	0
6-Methyl-5-hepten-2-one	0.32 ± 0.03	6.40	11.43	0	4.48	0	0	1.92	0
(*E*,*E*)-2,4-Heptadienal	0.28 ± 0.01	5.71	10.20	0	0	0	0	0	5.71
1-Pentanol	0.24 ± 0.02	1.60	2.86	0	0	0	0	0	1.6
1-Penten-3-ol	0.57 ± 0.02	1.59	2.84	0	0	0.64	0.32	0.63	0
(*E*)-Geranylacetone	0.27 ± 0.02	1.45	2.59	0	1.30	0	0	0	0.15
Acetic acid	7.61 ± 0.25	1.38	2.47	0	0	0	0	0.14	1.24
Hexanal	0.65 ± 0.07	1.36	2.43	0.14	0	0	0.13	0.27	0.82
Epoxydihydrolinalool	0.56 ± 0.01	1.12	2.00	1.09	0	0	0	0	0.03
Linalool	0.54 ± 0.04	1.08	1.93	0	1.08	0	0	0	0
Total	11.28	55.98 *		18.37	17.15	7.50	0.45	2.96	9.55
% **				32.81	30.64	13.40	0.80	5.29	17.06

*Note*: OAVi = Ci/Ti, where Ci is the content of compound i (μg kg^−1^), and Ti is the threshold value of compound i (μg kg^−1^). * OVAt = ∑OAVi. ** % is proportion of the each odor OAV accounting for OAVt.

## Data Availability

The data presented in this study are available on request from the corresponding author.

## Supplementary Materials

The following supporting information can be downloaded at: https://www.mdpi.com/article/10.3390/foods11152204/s1, Table S1: The threshold value and odor characteristics of some aroma compounds.; Table S2: The content of volatile compounds in tea with different types (μg kg^−1^) (n = 3).
