# Supplementary material for: Digital Evaluation of Aroma Intensity and Odor Characteristics of Tea with Different Types—Based on OAV-Splitting Method
Source: Foods. 2022 Jul 25;11(15):2204. doi: 10.3390/foods11152204 (PMC9329961; doi:10.3390/foods11152204)
Supplement: Supplementary file 1 [file foods-11-02204-s001.zip › foods-1814332-supplementary.pdf]

Supplementary materials

Table S1. The threshold value and odor characteristics of some aroma compounds.

Compound	Threshold value* ($\mu g\ kg^{-1}$)	Split of odor characteristic (%)**					
		Woody	Floral	Burnt	Green	Fruity	Fatty
3-Octanone	0.001	15	0	0	75	10	0
β-Cyclocitral	0.003	10	0	0	20	70	0
β-Ionone	0.007	50	30	20	0	0	0
Valeraldehyde	0.012	0	0	0	10	20	70
1-Octen-3-ol	0.04	0	0	80	10	0	10
Methyl salicylate	0.04	0	70	30	0	0	0
(*E*,*E*)-2,4-Heptadienal	0.049	0	0	0	0	0	100
6-Methyl-5-hepten-2-one	0.05	0	70	0	0	30	0
2(5H)-Furanone	0.06	0	10	10	0	60	10
(*E*)-3-Hexen-1-ol	0.07	0	0	0	90	10	0
1-Octanol	0.11	0	20	0	40	0	40
(*E*,*E*)-3,5-Octadien-2-one	0.15	0	0	100	0	0	0
1-Pentanol	0.1503	0	0	0	0	0	100
(*E*)-Geranylacetone	0.186	0	90	0	0	0	10
2-Hexen-1-ol	0.232	0	0	0	100	0	0
Furfural	0.282	0	0	80	0	0	20
1-Penten-3-ol	0.3581	0	0	20	20	40	0
Hexanal	0.479	10	0	0	10	20	60
Linalool	0.5	0	100	0	0	0	0
Epoxydihydrolinalool	0.5	97	0	0	0	0	3
Phenethyl alcohol	0.75	10	80	0	10	0	0
Benzaldehyde	3.5	5	0	0	10	75	10
Acetic acid	5.5	0	0	0	0	10	90

* Threshold Values were referred to ‘Compilations of Odour Threshold Values in Air, Water and other Media’ (Second Enlarged and Revised Edition). L.J. van Gemert. 2011[22,23].

** Split of odor characteristics were referred to Lin X.Y. Perfumery (3rd Edition). Chemical Industry Press. 2018[17].

Table S2. The content of volatile compounds in tea with different types (μg kg^{-1}) (n=3)

Compound	White tea	Green tea	Oolong tea	Red tea	Dark tea
Terpenoids					
Linalool	3.29±0.08	0.39±0.02	0.21±0.01	1.40±0.10	0.54±0.04
Epoxydihydrolinalool	2.91±0.26	0.13±0.01	0.37±0.01	/	0.56±0.01
3,7-Dimethyl-6-octen-3-ol	/	/	0.41±0.02	/	/
Linalool oxide (Pyranoid)	0.77±0.03	/	/	/	/
Terpineol	/	/	/	/	0.18±0.00
Safranal	/	0.21±0.01	0.14±0.00	0.57±0.07	0.24±0.06
β-Cyclocitral	/	0.30±0.05	0.36±0.01	0.79±0.09	/
β-Ionone	0.16±0.02	0.19±0.00	0.32±0.02	0.66±0.04	0.24±0.01
β-Ionon-5,6-epoxide	/	0.06±0.00	/	0.08±0.00	/
4-Oxoisophorone (tea ketone)	/	0.15±0.01	0.13±0.01	0.32±0.01	0.15±0.00
Isophorone	/	0.21±0.01	0.16±0.01	0.36±0.04	/
2,2,6-Trimethylcyclohexanone	/	0.60±0.04	0.26±0.03	0.55±0.03	0.18±0.01
(*E*)-Geranylacetone	/	/	0.09±0.00	/	0.27±0.02
Dihydroactindiolide	0.09±0.02	0.13±0.01	0.08±0.01	0.18±0.00	0.16±0.01
Limonene	/	0.16±0.01	0.34±0.08	0.74±0.08	/
Ocimene	/	/	0.81±0.14	/	/
(-)-α-Cedrene	0.10±0.02	/	/	0.19±0.01	/
(+)-Δ-Cadinene	/	0.14±0.00	0.81±0.13	/	/
Alcohol					
1-Penten-3-ol	0.20±0.03	5.04±0.25	2.59±0.12	0.08±0.01	0.57±0.02
(*E*)-3-Hexen-1-ol	0.73±0.01	1.54±0.07	0.15±0.00	1.52±0.02	/
2-Hexen-1-ol	/	0.31±0.01	/	/	/
2,6-Dimethylcyclohexanol	0.16±0.02	/	/	/	/
4-tert-Butylcyclohexanol	0.62±0.10	0.69±0.08	0.62±0.10	/	0.53±0.04
Phenol	/	/	/	0.05±0.01	0.06±0.01
Benzyl alcohol	1.63±0.12	0.54±0.02	0.09±0.00	1.91±0.20	0.42±0.02
Phenethyl alcohol	3.38±0.56	0.12±0.00	0.24±0.04	2.04±0.22	0.47±0.02
1-Phenethylalcohol	0.17±0.01	/	/	/	/
Furfuryl alcohol	/	0.15±0.01	0.39±0.07	0.27±0.01	0.20±0.02
1-Pentanol	/	0.79±0.01	/	1.61±0.13	0.24±0.02
1-Octen-3-ol	/	0.52±0.04	/	/	/
1-Octanol	/	0.13±0.02	/	/	/
2-Ethylhexanol	/	/	/	/	0.19±0.02
2,3-Butanediol	/	/	/	/	4.48±0.27
Aldehyde					
Valeraldehyde	/	0.67±0.08	0.80±0.12	/	/
Hexanal	0.84±0.01	1.39±0.02	3.29±0.10	1.57±0.01	0.65±0.07
Heptaldehyde	/	/	/	/	0.17±0.00
Nonanal	0.22±0.04	/	/	/	0.14±0.01
(*E*)-2-Pentenal	/	/	1.45±0.07	/	0.60±0.04
(*E*)-2-Hexenal	0.10±0.02	0.32±0.01	0.56±0.00	0.63±0.02	0.35±0.03
(*E*,*E*)-2,4-Heptadienal	/	0.25±0.00	0.71±0.09	/	0.28±0.01
(*E*)-2-Pentenal	/	/	0.96±0.10	/	/
2-Methyl-2-pentenal	/	0.21±0.00	/	/	/
Benzaldehyde	2.21±0.26	0.45±0.02	0.25±0.04	4.83±0.71	0.19±0.02
Furfural	/	/	2.06±0.06	4.62±0.41	/
5-Methyl furfural	/	/	0.18±0.04	3.11±0.35	/
Pyrrole-2-carboxaldehyde	/	/	/	0.25±0.00	/
N-Methylpyrrole-2-carboxaldehyde	/	/	/	0.29±0.05	/
Ketone					
Acetone	1.35±0.10	1.86±0.01	/	/	/
Hydroxyacetone	/	/	0.29±0.10	/	/
3-Hydroxy-2-butanone	/	/	/	/	0.38±0.02

3-Octanone	/	0.23±0.01	/	/	/
6-Methyl-2-heptanone	/	0.19±0.01	/	/	/
4-Methyl-3-penten-2-one	/	1.10±0.05	/	/	/
6-Methyl-5-hepten-2-one	0.47±0.01	0.71±0.06	1.54±0.19	1.83±0.23	0.32±0.03
6-Methyl-3,5-heptadien-2-one	/	0.15±0.01	0.14±0.00	0.44±0.03	/
3,5-Octadien-2-one	/	/	0.23±0.01	/	/
4-tert-Butylcyclohexanone	/	0.73±0.03	0.53±0.06	/	1.10±0.08
2(5H)-Furanone	/	/	/	0.26±0.02	/
Jasmone	/	0.10±0.00	/	/	/
Acetophenone	0.26±0.02	/	/	0.51±0.06	/
Acid					
Acetic acid	3.82±0.39	1.60±0.02	2.28±0.26	22.57±0.73	7.61±0.25
Propionic acid	1.18±0.04	0.33±0.01	0.24±0.00	2.45±0.07	0.48±0.03
Valeric acid	0.98±0.06	0.27±0.01	/	1.55±0.13	/
Hexanoic acid	2.86±0.19	0.60±0.01	0.12±0.01	3.10±0.35	0.18±0.00
Heptanoic acid	0.29±0.03	0.08±0.01	/	0.17±0.00	/
Octanoic acid	0.12±0.02	/	/	/	/
(*E*)-3-Hexenoic acid	0.22±0.01	/	/	0.14±0.02	/
(*E*)-2-Hexenoic acid	0.25±0.01	/	/	/	/
Ester					
Formic acid isopropyl ester	/	/	/	/	0.30±0.08
Hexyl acetate	/	/	/	0.20±0.02	/
Methyl hexanoate	/	0.97±0.13	0.24±0.02	0.54±0.05	/
Caprylic acid methyl ester	0.16±0.03	/	/	/	/
Methyl heptanoate	/	0.23±0.01	/	/	/
Methyl nonanoate	/	0.15±0.04	/	/	/
Methyl salicylate	0.27±0.02	/	/	0.41±0.05	/
Methyl phenylacetate	0.16±0.02	/	/	/	/
γ-Butyrolactone	/	/	/	2.34±0.05	/
4-Hexanolide	0.53±0.06	0.20±0.00	0.18±0.01	0.48±0.05	/
(*E*)-3-Hexenyl hexanoate	/	0.15±0.01	/	/	/
Pantolactone	/	/	/	/	0.09±0.01
Heterocycle					
2-Ethylfuran	0.09±0.00	1.41±0.23	1.18±0.10	/	/
2-Pentylfuran	0.22±0.03	2.91±0.48	0.86±0.17	1.58±0.34	0.20±0.02
2-(2-Pentenyl)furan	/	0.47±0.06	/	/	/
2-Acetylfuran	/	0.11±0.00	0.17±0.04	1.71±0.17	/
2-Acetyl pyrrole	0.23±0.01	0.07±0.00	0.08±0.03	0.72±0.07	0.07±0.00
2-Methylpyrazine	/	0.26±0.01	0.52±0.10	2.18±0.10	/
Ethylpyrazine	/	/	/	1.65±0.21	/
2,3-Dimethylpyrazine	/	0.28±0.01	/	0.18±0.01	/
2,5-Dimethyl pyrazine	0.19±0.05	/	/	/	/
2-Ethyl-3-methylpyrazine	/	/	/	0.36±0.04	/
2-Ethyl-5-methylpyrazine	/	/	0.18±0.00	0.37±0.05	/
3-Ethyl-2,5-dimethylpyrazine	/	0.15±0.03	/	/	/
Hydrocarbon					
Toluene	/	0.96±0.12	2.89±0.21	/	0.12±0.02
o-Xylene	/	/	1.41±0.12	/	/
o-Cymene	/	/	0.26±0.03	0.77±0.08	/
1,2,3-Trimethylbenzene	/	/	/	0.48±0.07	/
Styrene	/	/	/	/	0.20±0.01
1,1,6-Trimethyltetralin	/	/	0.14±0.00	0.38±0.05	/
Decane	/	0.59±0.03	0.22±0.02	/	/
Hendecane	/	0.86±0.11	/	/	/
Dodecane	/	/	0.52±0.04	/	/
Tridecane	/	0.37±0.01	/	/	/

Tetradecane	0.23±0.03	/	0.18±0.02	/	0.17±0.02
Pentadecane	/	/	/	0.65±0.10	/
Nonadecane	0.14±0.07	/	/	/	/
Eicosane	0.41±0.07	/	/	0.84±0.07	0.13±0.01
2,2,4,6,6-Pentamethylheptane	/	/	/	/	2.07±0.20
(*E*)-4,4-Dimethyl-2-pentene	/	/	/	0.37±0.00	/
Total	32.01	33.88	33.23	76.60	25.48

/ - no detection.
